# Association prediction of lncRNAs and diseases using multiview graph convolution neural network

**DOI:** 10.3389/fgene.2025.1568270

**Published:** 2025-04-15

**Authors:** Wei Zhang, Yifu Zeng, Xiaowen Xiang, Bihai Zhao, Sai Hu, Limiao Li, Xiaoyu Zhu, Lei Wang

**Affiliations:** ^1^ College of Computer Science and Engineering, Changsha University, Changsha, Hunan, China; ^2^ Department of Information and Computing Science, College of Mathematics, Changsha University, Changsha, China

**Keywords:** graph convolutional network, lncRNA-miRNA, multiview data, deep learning, similarity network

## Abstract

Long noncoding RNAs (lncRNAs) regulate physiological processes via interactions with macromolecules such as miRNAs, proteins, and genes, forming disease-associated regulatory networks. However, predicting lncRNA-disease associations remains challenging due to network complexity and isolated entities. Here, we propose MVIGCN, a graph convolutional network (GCN)-based method integrating multimodal data to predict these associations. Our framework constructs a heterogeneous network combining disease semantics, lncRNA similarity, and miRNA-lncRNA-disease interactions to address isolation issues. By modeling topological features and multiscale relationships through deep learning with attention mechanisms, MVIGCN prioritizes critical nodes and edges, enhancing prediction accuracy. Cross-validation demonstrated improved reliability over single-view methods, highlighting its potential to identify disease-related lncRNA biomarkers. This work advances network-based computational strategies for decoding lncRNA functions in disease biology and provides a scalable tool for prioritizing therapeutic targets.

## 1 Introduction

Recently, the rapid evolution of artificial intelligence has led to the widespread implementation of deep learning algorithms across various fields, such as computer vision, recommendation systems, and interdisciplinary areas such as bioinformatics (Guzman-Pando et al., 2024; Piroozmand et al., 2020; Zhou et al., 2023). In contrast to conventional machine learning techniques, deep learning frameworks are capable of constructing complex information propagation architectures that facilitate the effective interpretation of diverse data types, demonstrating superior learning and representation abilities. In the domain of relation prediction between lncRNAs and diseases, deep learning methodologies have been shown to surpass conventional approaches by more accurately extracting intricate and nuanced features. Recently, many deep learning-based methods, including autoencoders (AEs), CNNs, graph convolutional networks (GCNs), and GANs, have been extensively utilized for predicting relationships between lncRNAs and diseases (Jin et al., 2021; Ha et al., 2020; Chen et al., 2021).

Wu et al. introduced an algorithm termed GAMCLDA, which is based on the principles of graph encoder matrix completion (Wu et al., 2020). This model effectively incorporates a wide range of biological data. The initial feature vectors for lncRNAs and diseases are derived from association data linking lncRNAs to diseases, genes, and microRNAs (miRNAs). A multilayer perceptron (MLP) is subsequently employed to reduce the dimensionality of these initial input features. To obtain the relevant structural characteristics of the lncRNA‒disease association network, a GCN is utilized, yielding feature vectors for both diseases and lncRNAs. The inner product of these two vectors serves as the predictive value for lncRNA‒disease associations. This approach constitutes a supervised deep learning model, with its efficacy largely contingent on the formulation of the loss function. To address the imbalance in two kinds of samples in lncRNA‒disease associations, the authors incorporate a learnable weight parameter into the loss function.

Zhao et al. also proposed an algorithm called MHRWR based on RWR. This method first constructs a three-layer heterogeneous network of lncRNA-disease-gene, then uses random walk to extract network structural features, and finally predicts associations (Zhao et al., 2021). In addition, Lu et al. also proposed a method called SIMCLDA based on inductive matrix completion (Lu et al., 2018). This method uses the association vectors of lncRNA diseases to calculate the GIP similarity between vectors, calculates the functional similarity of diseases based on the association data of disease genes, and then constructs the feature vectors. PCA was then used to reduce the lncRNA and disease feature vector dimensions. Finally, the incidence matrix was reconstructed based on the induction matrix. In order to make full use of the structural information between lncRNAs disease association matrices, Lu et al. proposed a lncRNAs disease association prediction algorithm called GMCLDA (Lu et al., 2020). The algorithm first calculated the semantic similarity of diseases, the GIP similarity of lncRNAs and diseases, and the gene sequence similarity of lncRNAs. Then, the K-nearest neighbor (KNN) algorithm based on the sequence similarity of lncRNAs updates its correlation matrix, and finally, matrix completion is used to reconstruct its correlation matrix.

Xuan et al. constructed a lncRNA‒disease association prediction model that employs a two-way CNN with an attention mechanism, referred to as cnnlda (Xuan et al., 2019a). This network integrates various data types, including similarity data for lncRNAs and diseases, lncRNA‒disease association data, and their relationships with miRNAs. The model is bifurcated into two components: the first component utilizes a CNN to extract characteristics directly from node pairs, and the second component acknowledges that different components of node pair features among lncRNAs, miRNAs, and disease nodes contribute variably to association prediction. This component assigns weight values to distinct types of association features and feature components prior to the convolutional layer.

Additionally, Xuan et al. constructed a CNN-based method named ldapred (Xuan et al., 2019b). This method initially constructs feature vectors for diseases and lncRNAs on the basis of the functional similarity matrix of lncRNAs, the semantic association matrix of diseases, and the functional similarity matrix of miRNAs. These feature vectors are then linked and input into a wide CNN to predict the output value. To effectively capture the topological information within the network, this method employs the concept of information dissemination, which uses second-order similarity matrices and association matrices for lncRNAs, diseases, and miRNAs during the computation of initial features. Furthermore, the method incorporates a two-way convolutional neural network to enhance model performance.

Graph convolutional networks (GCNs) represent an important method for extracting structural features from graph data (Wang et al., 2024). In recent years, GCNs have found extensive applications in various domains, including node prediction, graph embedding representation, and graph classification. In the field of bioinformatics, GCNs are frequently employed for link prediction, because the regulatory relationships among biomacromolecules are often represented as structured graphs.

Xuan et al. introduced a disease association prediction model that integrates GCN and CNN techniques (Xuan et al., 2019c), termed GCNLDA. This method constructs heterogeneous graphs representing miRNA and lncRNA diseases, utilizing a GCN to extract topological features from these graphs to derive feature vectors for the nodes. A simple neural network is subsequently employed to predict the correlations between diseases and increases. Additionally, the model leverages a CNN to capture the characteristics of node pairs for correlation prediction, fusing the prediction scores through a weighted approach.

Furthermore, acknowledging the varying contributions of distinct subgraphs within heterogeneous graphs to the prediction process, Zhao et al. proposed a meta-path-based disease association prediction model for lncRNAs, referred to as Heated (Zhao et al., 2022). This model segments the lncRNA‒disease heterogeneous network into five subgraphs: disease‒lncRNA‒disease, lncRNA‒disease, lncRNA‒disease‒lncRNA, lncRNA‒disease‒lncRNA, and lncRNA‒disease. Feature vectors are derived from these five subgraphs via a graph attention network (GAT) (Dai et al., 2022), and weighted values are utilized to integrate the feature vectors corresponding to different lncRNAs and diseases. The model concludes by employing a neural network matrix completion algorithm to predict the association matrix.

Moreover, several researchers have achieved enhanced prediction outcomes by integrating machine learning models with deep learning frameworks. Jihwan Ha et al. provided a framework based on deep neural networks for predicting miRNA-disease associations (NCMD) known as Neural Collaborative Filtering based on node2vec. NCMD leverages node2vec to learn low-dimensional vector representations of miRNAs and diseases. It utilizes a deep learning framework that combines the linear capability of generalized matrix factorization with the nonlinear capability of multilayer perceptrons (Ha and Park, 2023). By applying a recommendation algorithm with miRNA and disease similarity constraints, Jihwan Ha et al. proposed a simple yet effective computational framework (SMAP) to identify associations between miRNAs and diseases. It measures comprehensive and accurate similarity values based on miRNA functional similarity, disease semantic similarity, and Gaussian Interaction Profile Kernel similarity. SMAP not only utilizes known miRNA-disease associations to construct a matrix factorization model but also incorporates the integrated similarity between miRNAs and diseases (Ha, 2023). Sheng et al. constructed an association prediction model named VADLP (Sheng et al., 2021), which constructs a heterogeneous network encompassing lncRNAs, diseases, and miRNAs. VADLP extracts network topology features of node pairs through a random walk algorithm and derives distribution features on the basis of a CNN encoder and variational autoencoder. These features are then adaptively fused to predict lncRNA‒disease relationships. Wu et al. introduced a prediction method based on random forests, termed GEAR (Wu et al., 2021). This model initially acquires feature representations of network nodes from the lncRNA–disease–miRNA heterogeneous network via a graph autoencoder, subsequently concatenating lncRNA and disease features to form node pair features. A random forest is then employed to investigate the potential associations between lncRNAs and disease. LAN et al. constructed a lncRNA‒disease relation prediction model utilizing a graph attention network, referred to as GANLDA (Lan et al., 2022). This approach inputs association vectors of increases, diseases, and genes, applies the principal component analysis (PCA) method (Zhang and Castelló, 2017) for dimensionality reduction, and utilizes the GAT to extract potential disease vectors, predicting correlations through a multilayer perceptron.

These studies collectively demonstrate the trend of combining graph-based structural analysis (GCN, GAT, random walks) with deep learning techniques (CNN, MLP, matrix completion) and multi-modal data fusion (functional similarities, semantic associations, sequence data). They increasingly focus on addressing data imbalance, enhancing feature representation through attention mechanisms, and improving prediction accuracy by exploiting heterogeneous network topologies and multi-source biological data integration.This paper proposes a graph convolutional network (GCN)-based method for predicting lncRNA-disease associations, grounded in the regulatory networks of biomolecules and diseases. Given that microRNAs (miRNAs) and long non-coding RNAs (lncRNAs) often cooperatively regulate gene expression, we construct a heterogeneous lncRNA-miRNA-disease interaction network. Furthermore, to address the issue of isolated diseases and lncRNAs lacking contextual relationships, we integrate disease semantic similarity networks and lncRNA similarity networks into the heterogeneous framework. Our contribution is a novel lncRNA-disease association prediction model called MVIGCN, which combines multi-view data sources with graph deep learning enhanced by attention mechanisms. This approach synergizes structural features extracted from the heterogeneous network with attention-weighted feature fusion, enabling more accurate and interpretable predictions of lncRNA-disease correlations.

## 2 Methods

The framework of the MVIGCN model has been introduced. Next, the details of the model, calculation methods, and loss functions are introduced one by one.

**FIGURE 1:**
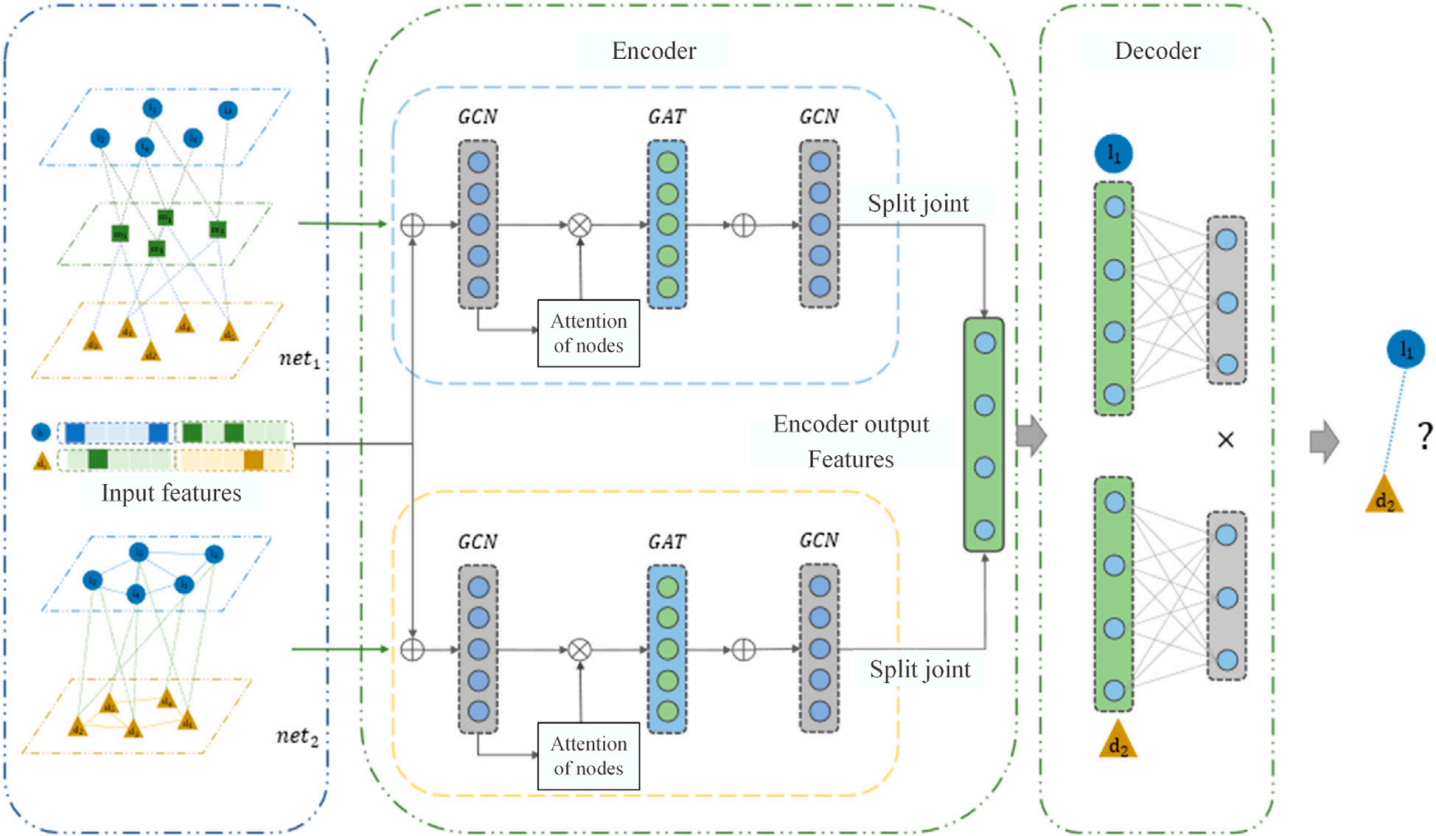
Diagram of the MVIGCN model structure.

MVIGCN model is a neural network model of codec structure, as shown in Figure 1, which mainly includes two parts: encoder part and decoder part. In this paper, the encoder was used to extract biological network features, eliminate data noise, and reduce feature dimensions, and the decoder was used to predict lncRNA disease association. The encoder is divided into two parts in the model, as shown in Figure 1. The node embedding vectors of two different graphs were learned separately. Specifically, the upper part of the encoder was learned by the node features of the graph convolutional neural network layer, and the lower part also used the node features of the graph convolutional neural network layer to learn the node features. The decoder part predicts the lncRNA disease association probability based on the latent features of the nodes.

### 2.1 Initial eigenvector construction

In this work, first, on the basis of the lncRNA‒miRNA association matrix, a three-layer heterogeneity map of lncRNA‒miRNA–disease ( ${net}_{1}$ ) was established by the lncRNA, disease similarity matrix, and lncRNA disease relation matrix, and the lncRNA disease relation graph ( ${net}_{2}$ ) was constructed. According to the hypothesis that “functionally similar lncRNAs tend to be related to diseases with similar phenotypes,” the MVIGCN model predicts associations by extracting the association features and similarity features of diseases and lncRNAs in the network. In the coding phase, the MVIGCN model selects lncRNAs, disease similarity features, and correlation features as its initial coding vector.

**FIGURE 2:**
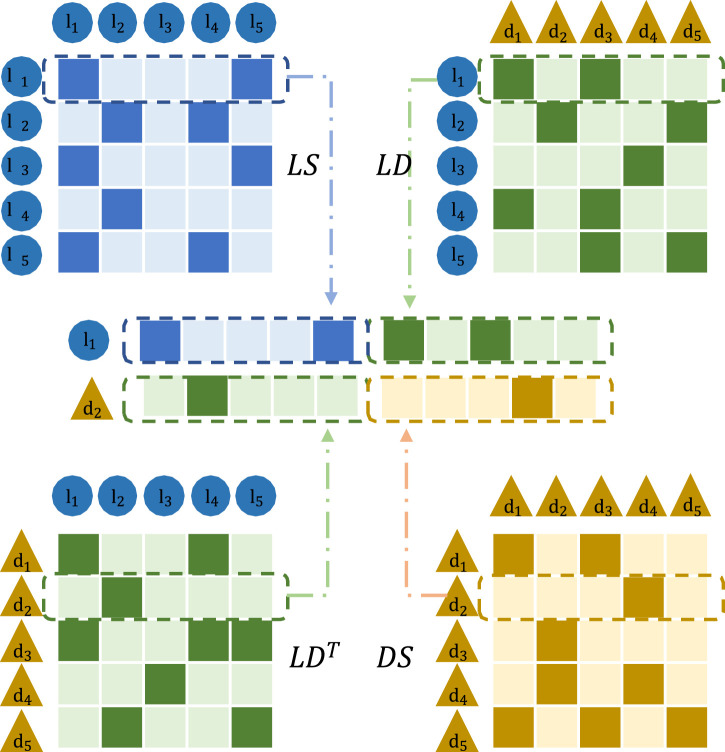
LncRNA and initial input eigenvectors of diseases.

Figure 2 shows the construction method of the initial input eigenvectors of $l_{1}$ and $d_{2}$ . The initial input feature of $l_{1}$ is ${feature}_{l1}=\left[LS\left(1,:\right);LD\left(1,:\right)\right]$ , where $LS\left(1,:\right)$ is the first line of the lncRNA similarity matrix LS and $LD\left(1,:\right)$ is the first line of the disease association matrix LD . The initial input characteristic ${feature}_{d2}=\left[{LD}^{T}\left(2,:\right);DS\left(2,:\right)\right]$ of $d_{2}$ , where ${LD}^{T}\left(2,:\right)$ is the second line of the LD transposition matrix and $DS\left(2,:\right)$ is the second line of the disease similarity matrix. On the basis of the above method, the initial characteristic matrix $F_{1}\in R^{\left(nl+nd\right)\times \left(nl+nd\right)}$ of lncRNAs and diseases is obtained. As shown in Equation 1, the definitions of a characteristic matrix $F_{1}$ and offset matrix $A_{2}$ of ${net}_{2}$ are the same. The range of $F_{1}\in R^{652\times 652}$ . Each line vector of $F_{1}$ represents the characteristic vector of the lncRNA or disease. For example, $F_{1}\left(1,:\right)$ is the characteristic vector of $l_{1}$ , and $F_{1}\left(241,:\right)$ is the characteristic vector of $d_{1}$ .

$$F_{1}=A_{2}=\left[\begin{array}{cc} LS & LD\\ {LD}^{T} & DS \end{array}\right]$$ (1)

### 2.2 Graph convolution neural networks

The conventional convolutional neural network (GNN) has demonstrated considerable efficacy in image processing and various other domains, effectively capturing informative features by extracting structured data, such as images and text (Sarıgül et al., 2019). However, unstructured data are prevalent across numerous fields, including social relationship mapping, citation analysis, and chemical molecular structure representation. Traditional convolutional operations require that data be translation invariant, a criterion that unstructured data often fail to satisfy. In this context, the degree of each node within a graph and the interconnections among nodes exhibit significant variability. To address these challenges, (Bianchi et al., 2022) introduced the graph convolutional network (GCN), a deep learning algorithm designed for graph data that employs convolutional operations tailored to graph structures. The GCN typically uses the graph as input to derive the eigenvectors of individual nodes by integrating the eigenvectors of neighbouring nodes as well as the topological information of the graph. Recently, GCNs have been applied extensively in areas such as text classification, recommendation systems (Kejani et al., 2020), and relationship extraction.

The methodologies associated with GCNs can markedly improve the performance of web-based predictive tasks, including drug‒disease correlation prediction, user‒item correlation prediction, and miRNA‒disease correlation prediction. The GCN is capable of aggregating information from adjacent nodes, resulting in similar eigenvectors for these nodes. By stacking multiple layers of the CNN, the model is able to learn higher-order associations among nodes, therefore capturing the topological characteristics of the graph.

Next, we use the lncRNA disease association network ( ${net}_{2}$ ) as an example to explain how to calculate the GCN. Assume that $A_{2}$ is the offset matrix of ${net}_{2}$ , that ${net}_{2}$ contains N_l lncRNAs and N_d diseases, and that X is the node eigenvector on ${net}_{2}$ . The Laplacian symmetric normalization matrix $L_{2}$ of Figure ${net}_{2}$ is defined as shown in Equation 2:

$$L_{2}=D_{2}^{-1/2}A_{2}D_{2}^{-1/2}$$ (2)

where $D_{2}$ is the degree matrix of graph ${net}_{2}$ , which describes the degree of each node, and $D_{2}$ is a diagonal matrix. The values of the diagonal elements are defined as shown in Equation 3:

$${\left[D_{2}\right]}_{ij}=\sum_{j}{\left[A_{2}\right]}_{ij}$$ (3)

The eigendecomposition of the $L_{2}$ matrix is carried out as shown in Equation 4:

$$L_{2}=U_{2}\Lambda_{2}U_{2}^{T}$$ (4)

where $U_{2}$ is the corresponding characteristic vector matrix, $\Lambda_{2}$ is the eigenvalue matrix, which is defined as $\Lambda_{2}=diag\left(\lambda_{1},\lambda_{2},\lambda_{3},...,,\lambda_{N}\right)$ , $\lambda_{i}$ is its eigenvalue, and $N=N_{l}+N_{d}$ . The GFT of the graph signal $X^{\left(t-1\right)}$ in Figure ${net}_{2}$ is transformed into a new graph semaphore $X^{\left(t\right)}$ via the following formula:

$$X^{\left(t\right)}=U_{2}\Lambda_{2}U_{2}^{T}X^{\left(t-1\right)}$$ (5)

where $\Lambda_{2}$ is regarded as the graph signal filter. In Equation 5, the graph semaphore $X^{\left(t-1\right)}$ is converted to the graph domain signal $X^{\prime \left(t-1\right)}=U_{2}^{T}X^{\left(t-1\right)}$ . After a filter is applied to the graph domain, the graph domain signal is inversely converted to the graph signal through $U_{2}X^{\prime \left(t-1\right)}$ . In Equation 5, filter $\Lambda_{2}$ is parameterized. After the parameters are introduced, the optimal filter can be learned in a supervised way. On the basis of the parameterized filter, the GFT can adjust the importance of each spectral domain in graph signal conversion. Hence, the filter is modified to ABC, and Equation 5 is rewritten as shown in Equation 6:

$$X^{\left(t\right)}=U_{2}\left({\Theta \Lambda }_{2}\right)U_{2}^{T}X^{\left(t-1\right)}$$ (6)

Nevertheless, Equation 6 has two significant limitations. First, in the context of large networks, the computation of the eigenvector and eigenvalue matrices is resource intensive. Second, the representation of each node by a singular scalar feature is inadequate for capturing the intricate and nuanced relationships among nodes. To address the first issue, Zhang et al. employed the first-order Chebyshev polynomial to approximate the filter (Zhang et al., 2024), subsequently reformulating Equation 6 as shown in Equation 7:

$$X^{\left(t\right)}={\tilde{D}}_{2}^{-1/2}{{\tilde{A}}_{2}{\tilde{D}}_{2}^{-1/2}X}^{\left(t-1\right)}\Theta_{1}$$ (7)

To enable nodes to retain their characteristics during feature propagation, the method adds node self-connected edges in ${net}_{2}$ and its corresponding adjacency matrix ${\tilde{A}}_{2}=A_{2}+I_{2}$ , where $I_{2}$ refers to the identity matrix and ${\tilde{D}}_{2\;}$ represents the degree matrix ${\left[{\tilde{D}}_{2}\right]}_{ii}=\sum_{j}{\left[{\tilde{A}}_{2}\right]}_{ij}$ of nodes. $\Theta_{1}$ is the first term of Θ and a scalar parameter. In this way, the eigendecomposition of ${net}_{2}$ is not needed, and the filter parameter size is reduced, thus accelerating the training process. The second problem can be solved by expanding the scalar graph signal X to the vector signal $X=R^{N*fm}$ . In addition, the vector filter parameter Θ is expanded to the parameter matrix $W_{c}\in R^{fm*F}$ , which represents that there are F filters and fm input channel filter matrices. The final spectral convolution is calculated as shown in Equation 8:

$$X^{\left(t\right)}={\tilde{D}}_{2}^{-1/2}{{\tilde{A}}_{2}{\tilde{D}}_{2}^{-1/2}X}^{\left(t-1\right)}W_{c}^{t-1}$$ (8)

Equation 8 is a feedforward linear neural network. To enhance the expression property of the model, the nonlinear activation function and offset matrix are introduced in this paper. The graph convolution calculation method in this paper is as shown in Equation 9:

$$X^{\left(t\right)}=relu\left({\tilde{D}}_{2}^{-1/2}{{\tilde{A}}_{2}{\tilde{D}}_{2}^{-1/2}X}^{\left(t-1\right)}W_{c}^{t-1}+B_{\;c}^{t-1}\right)$$ (9)

where $B_{2}^{t-1}\in R^{N*F}$ refers to the offset matrix, $relu\left(\cdot \right)$ represents the nonlinear activation function, and $X^{\left(0\right)}$ in this paper is the initial feature vector.

**FIGURE 3:**
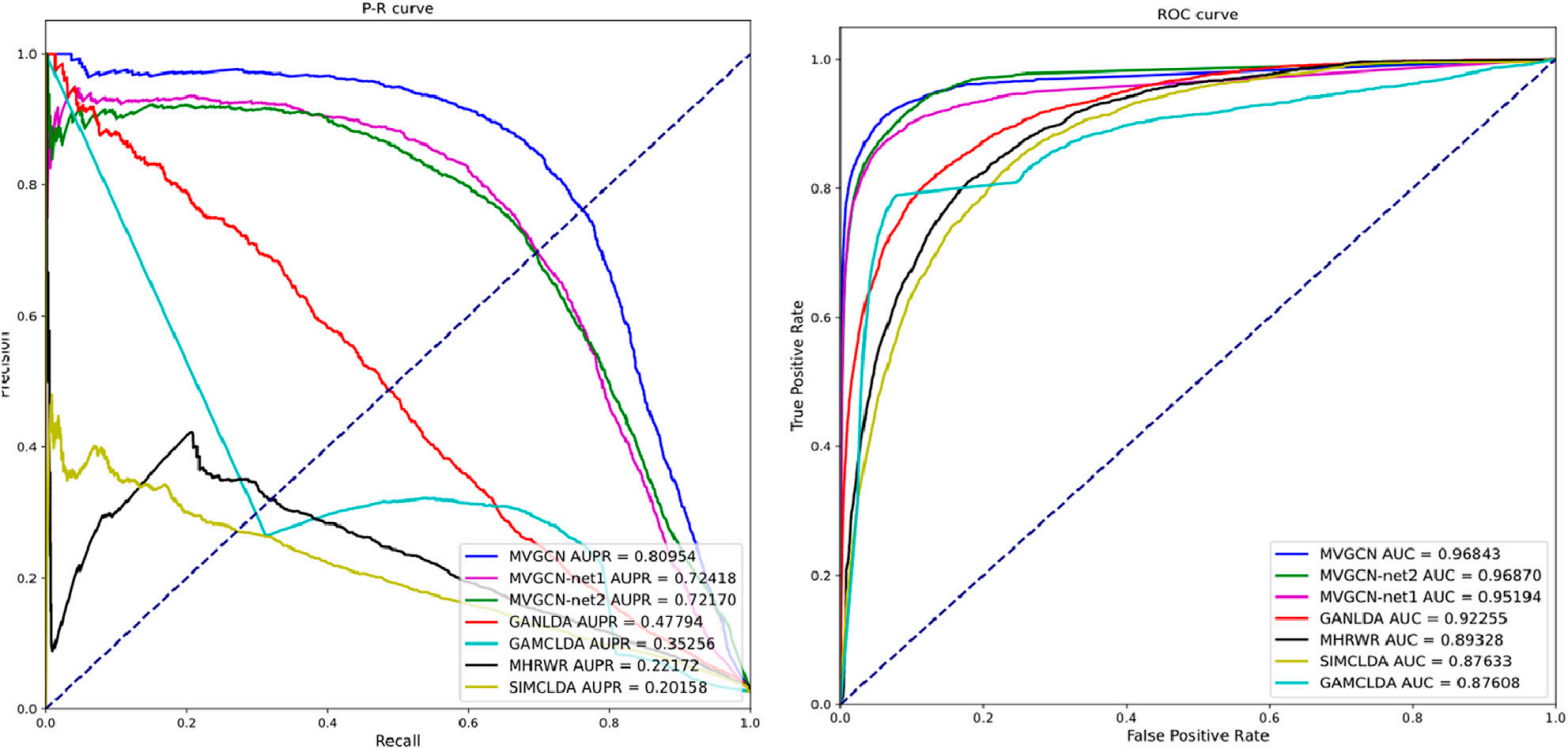
ROC curve and P-R curve of MVIGCN model and comparison model.

As shown in Figure 3, this paper uses a GCN to extract the features of ${net}_{1}$ and ${net}_{2}$ , uses $F_{1}$ as the input, and uses a GCN to extract the features of ${net}_{1}$ and ${net}_{2}$ . Assume that the feature vector matrices of the two output nodes are $H_{1}^{net1}$ and $H_{1}^{net2}$ .

### 2.3 Graph attention network

Recently, attention mechanism-based deep learning models have received extensive attention. The attention mechanism is often applied in sequence tasks, which allows models to focus on key parts of the input. For example, in a machine reading task based on a recursive neural network (RNN), the model employs an attention mechanism to extract the key features of sentences and improve their sentence representation learning ability. Chan TH et al. applied the attention mechanism to graph node-embedded learning and then calculated a reasonable feature representation of nodes. This model is referred to as the GAT (Chan et al., 2024). The GAT, which can be considered an extension of the GCN, assigns different weights to neighbours through the self-attention layer and combines the features of neighbours. This operation can filter the noise in the network, concentrate on the more important correlation, and then extract the graph structure features effectively. Recently, the GAT has attracted widespread attention in the fields of node classification, social impact analysis, and recommendation systems because of its powerful graph feature extraction capability. ${net}_{2}$ is used as an example to explain how to calculate the GAT proposed by Liu et al. (Qiu et al., 2024).

Given a node, the GAT needs to calculate the importance of its neighbours. Using node i as an example, first, the attention score of its neighbour is calculated. Equation 10 expresses the attention score of node j on node i :

$$e_{ij}^{2}={\left(W_{t}h_{j}\right)}^{T}\tan h\left(\left(W_{t}h_{i}+b\right)\right)$$ (10)

In the formula, $h_{i}$ and $h_{j}$ represent the characteristic representations of node i and node j , respectively, and $W_{t}$ and b refer to the trainable weight matrix and offset matrix, respectively. Second, the attention score is standardized and is shown in Equation 11:

$$\alpha_{ij}^{2}=\frac{\exp\left(e_{ij}^{2}\right)}{\sum_{k\in N_{i}^{2}}\exp\left(e_{ik}^{2}\right)}$$ (11)

where $N_{i}^{2}$ refers to the neighbour number of node i in ${net}_{2}$ . Last, different weight values are used to merge the features of neighbours. The eigenvector of final node i is expressed as shown in Equation 12:

$$h_{i}^{\prime }=\sigma \left(\sum_{j\in N_{i}^{2}}\alpha_{ij}^{2}\cdot h_{j}\right)$$ (12)

where σ represents the activation function, such as ReLU .

As shown in Figure 3, after the GCN layer, the MVIGCN model introduces the above GAT layer to update the node feature representation. The GAT uses $H_{1}^{net1}$ and $H_{1}^{net2}$ as inputs, and on the basis of the above calculation method, the corresponding potential feature matrices $H_{2}^{net1}$ and $H_{2}^{net2}$ are obtained. To extract the complex associations between nodes, the GCN layer is added to the model after the GAT, and the potential eigenvector matrices $H_{3}^{net1}\in R^{N\times l1}$ and $H_{3}^{net2}\in R^{N\times l2}$ of nodes ${net}_{1}$ and ${net}_{2}$ are obtained. The encoder calculation formula is as shown in Equations 13, 14:

$$H_{3}^{net1}=GCN\left(GAT\left(GCN\left(F_{1}\right)\right)\right)$$ (13)

$$H_{3}^{net2}=GCN\left(GAT\left(GCN\left(F_{1}\right)\right)\right)$$ (14)

The two potential eigenvector matrices are spliced to obtain the encoder output $Y\in R^{N\times \left(l1+l2\right)}$ , and the calculation formula is as shown in Equation 15:

$$Y=\left[\begin{array}{c} Y_{l}\\ Y_{d} \end{array}\right]=\left[\begin{array}{cc} H_{3}^{net1} & H_{3}^{net2} \end{array}\right]$$ (15)

where $Y_{l}\in R^{N\_l\times \left(l1+l2\right)}$ and $Y_{d}\in R^{N\_d\times \left(l1+l2\right)}$ are potential vectors of lncRNAs and diseases, respectively.

### 2.4 Bilinear decoder

The MVIGCN model uses a bilinear decoder to reconstruct the association. The calculation formula is as shown in Equations 16, 17:

$${LD}^{\prime }=Y_{l}W_{l}{\left(Y_{d}W_{d}\right)}^{T}$$ (16)

$$L_{REC}=\sum_{\left(i,j\right)\in P\cup N}\Theta \left({LD}_{ij}^{\prime },{LD}_{ij}\right)$$ (17)

where ${LD}^{\prime }\in R^{N\_l\times N\_d}$ is the probability matrix of such associations predicted by the decoder. For each element in ${LD}^{\prime }$ , the greater the value of ${LD}_{ij}^{\prime }$ is, the stronger the association between lncRNA i and disease j , and the weaker the correlation otherwise. $W_{d}\in R^{\left(l1+l2\right)\times r}$ and $W_{l}\in R^{\left(l1+l2\right)\times r}$ are trainable parameters used to map the learned potential features back to the initial feature space of the lncRNA and disease, $L_{REC}$ is the loss function of the reconstruction error, and $\Theta \left(\cdot \right)$ is the loss function of the classification, such as the mean square error (MSE loss). P and N refer to two kinds of sample training sets.

In association prediction model training, known associations are usually regarded as positive samples, whereas unknown associations are regarded as negative samples. However, in most cases, the relationship between lncRNAs and disease is unknown. The number of positive samples in training is far greater than the number of negative samples in training, and the data of positive samples and negative samples are extremely unbalanced. The model tends to predict all the correlation values as 0 so that a lower loss value can be obtained. However, the model cannot effectively distinguish positive samples. A cost-sensitive neural network was applied to address this issue (Zhang et al., 2019). The cost-sensitive loss function has been widely used in unbalanced learning. The modified reconstruction error loss function is as shown in Equation 18:

$$L_{REC}=\alpha {\left\|\Omega_{P}\left({LD}^{\prime }-LD\right)\right\|}_{F}^{2}+\left(1-\alpha \right){\left\|\Omega_{N}\left({LD}^{\prime }-LD\right)\right\|}_{F}^{2}$$ (18)

where $\Omega_{P}\in {\left\{0,1\right\}}^{N\_l\times N\_d}$ is the positive sample mask matrix of the lncRNA disease association data, $\Omega_{N}\in {\left\{0,1\right\}}^{N\_l\times N\_d}$ is the negative sample mask matrix, and α is the positive and negative sample balance parameter. In this work, a greater weight is allocated to the loss of positive samples, and a smaller weight is allocated to the loss of negative samples. The calculation method is as shown in Equation 19:

$$\alpha =\frac{\sum_{ij}\Omega_{P,ij}}{\sum_{ij}\left(\Omega_{P,ij}+\Omega_{N,ij}\right)}$$ (19)

In the training of a deep learning model, it is usually assumed that the training and test sets have the same distribution and that the model is trained in the training set space. However, sometimes, the distributions of the two sets are not the same. If the model is overfitted on the training set, its performance on the test set will be reduced. To increase the generalizability, regularization terms are usually added to the loss function. The parameters $W_{d},W_{c},W_{t}$ and B in the MVIGCN model affect the model generalizability. Therefore, the loss of the regularization term is defined as shown in Equation 20:

$$L_{\Omega }={\left\|W_{d}\right\|}^{2}+{\left\|W_{c}\right\|}^{2}+{\left\|W_{t}\right\|}^{2}+{\left\|B\right\|}^{2}$$ (20)

Therefore, the total loss $L_{Total}$ can be determined as shown in Equation 21, where γ is a weight factor:

$$L_{Total}=L_{REC}+\gamma L_{\Omega }$$ (21)

This research employs the Adam optimizer to train the MVIGCN model.

## 3 Results

### 3.1 Performance assessment of the MVIGCN model

This study employs a 50 percent cross-validation methodology to assess the properties of the model. The dataset, comprising both types of samples, is randomly partitioned into five subsets. One subset is designated as the test set, while the remaining subsets are designated as the training set. The model is trained on the training set, and its performance is subsequently evaluated on the test set. The evaluation outcomes from the test set are then aggregated to form a comprehensive dataset, and the predictive results are analyzed using various evaluation metrics, such as the TPR (true positive rate), FPR (false positive rate), Precision (precision), and Recall (recall rate) (Chen et al., 2021). The parameters involved in the model are set as follows: ∆ is the semantic decay factor, which is set to 0.5; $\gamma_{d}$ is the hyperparameter controlling the kernel width, set to 1; $\gamma_{l}^{\prime }$ is the normalized kernel bandwidth adjustment factor, set to 1; DCOS is the cosine similarity matrix between 412 diseases, with elements ranging from 0 to 1; $\alpha_{1}$ and $\alpha_{2}$ represent the weight values of the Gaussian interaction kernel similarity and cosine similarity values of lncRNA, respectively; $\beta_{1}$ and $\beta_{2}$ represent the weight values of the two types of similarities for diseases; $\alpha_{1}$ , $\alpha_{2}$ , $\beta_{1}$ , and $\beta_{2}$ are set to 0.1, 0.9, 0.2, and 0.8, respectively.

Furthermore, this research compares the proposed model against several existing models, including GAMCLDA (Mishra et al., 2020), which is predicated on graph convolutional neural networks and matrix decomposition; GANLDA (Li et al., 2021), which uses a graph attention network; MHRWR (Zhao et al., 2021), which is based on random walk principles; and SIMCLDA (Lu et al., 2018), which is founded on matrix completion. To further substantiate the impact of the multiview characteristics on the association prediction model, the study incorporates relevant ablation experiments. The MVIGCN-net1 model uses only the lncRNA‒miRNA–disease heterogeneous network for prediction, whereas the MVIGCN-net2 model relies on the association network. The ablation experimental model employs the same graph deep learning network architecture as the MVIGCN to learn lncRNA‒disease associations. The results from both the comparative analysis and the ablation experiments are presented in Table 1. Additionally, ROC and PR curves are generated to show the differences in predictive performance among the models.

**TABLE 1:** Experimental results of MVIGCN model and comparison model.

	AUC	AUPR	$F_{1}$ -score	MCC
MVIGCN	**0.9684**	**0.8095**	**0.7663**	**0.7640**
MVIGCN-net1	0.9519	0.7242	0.6781	0.6712
MVIGCN-net2	**0.9687**	0.7217	0.6525	0.6487
GAMCLDA	0.8761	0.3526	0.1525	0.2083
GANLDA	0.9226	0.4779	0.2797	0.3652
MHRWR	0.8933	0.2217	0.3106	0.3193
SIMCLDA	0.8763	0.2016	0.1103	0.1688

BMI; body mass index. Age and BMI, are means with range presented in parentheses.

* The results of this paper and the better performance of other methods are highlighted in bold.

As shown in Table 1 and Figure 3, the MVIGCN model presented in this study outperforms the four other correlation prediction models. Furthermore, the results of the experiment underscore the importance of the multiview approach utilized by the MVIGCN model. The data indicate that the AUC value for the MVIGCN model is 0.96843, which markedly exceeds the values of GANLDA (0.92255), MHRWR (0.89328), SIMCLDA (0.87633), and GAMCLDA (0.87633). In terms of the recall and accuracy rates, the recall rate serves as an indicator of the capacity of the model to predict positive samples, whereas the accuracy rate reflects the reliability of the model in predicting these samples. An effective model typically maximizes both the number of accurately predicted positive samples and the accuracy of those predictions; however, these two metrics cannot be optimized simultaneously. The AUPR and score serve as comprehensive metrics for evaluating both parameters concurrently. The AUPR value for the MVIGCN model is 0.80954, significantly surpassing the values of GANLDA (0.47794), GAMCLDA (0.35256), MHRWR (0.22172), and SIMCLDA (0.20158). Additionally, the score of the MVIGCN model is considerably higher than those of the other four models, indicating its robust performance on imbalanced datasets. With respect to the MCC evaluation index, the MVIGCN model outperforms all other models, highlighting its substantial advantages in predicting these associations.

In the ablation experiment, the MVIGCN model exhibited notable performance enhancements compared with MVIGCN-net1 and MVIGCN-net2, with the multiview fusion model demonstrating superior results across the AUC, AUPR, MCC, and other metrics. As depicted in Figure 1, the AUC value for the MVIGCN model is 0.9684, showing no significant improvement relative to MVIGCN-net1 (0.9519) and MVIGCN-net2 (0.9687). However, for the other metrics, the AUPR values for MVIGCN-net1 and MVIGCN-net2 are 0.7242 and 0.7217, respectively, while their scores are 0.6781 and 0.6525, and their MCC scores are 0.6712 and 0.6487, respectively, which are inferior to the performance of the MVIGCN model. These findings indicate that the constructed prediction model, which is based on multiple related data sources, plays a key role in improving the predictive efficacy of the model and addressing the challenges posed by imbalanced samples.The ablation experiments demonstrate that:Feature alignment and cross-view attention are critical for leveraging multi-view heterogeneity.Graph convolutional layers are indispensable for modeling biological network topology.Noisy views can be mitigated through learned attention, highlighting the model’s robustness.This analysis not only validates the MVIGCN’s design but also provides actionable insights for improving similar architectures in complex graph-based tasks.

**FIGURE 4:**
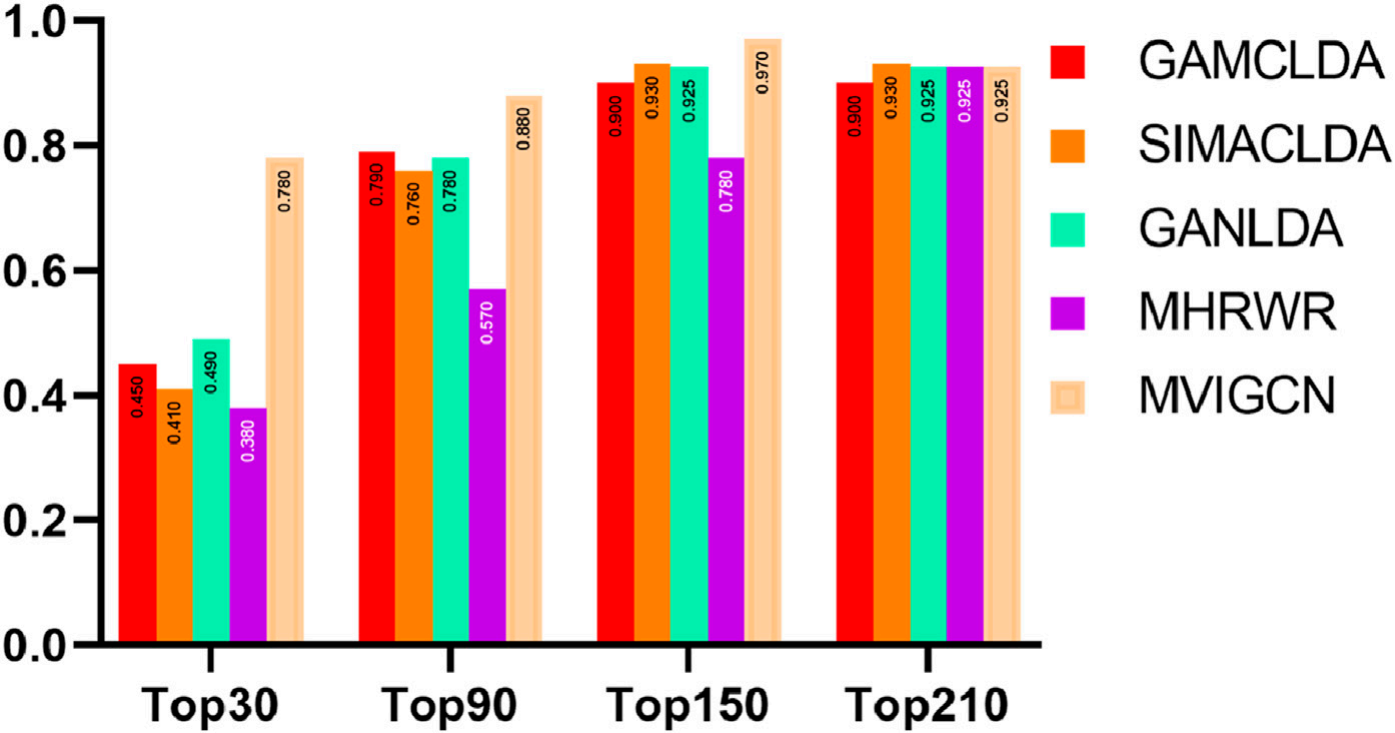
Top-k recall rate of MVIGCN model and comparison model.

The recall rate serves as an indicator of the capacity of a model to predict potential correlations and is a critical metric in secondary classification tasks. Importantly, the recall rate can fluctuate significantly across different probability thresholds. Moreover, establishing a consistent and appropriate threshold for calculating the recall rate across various correlation prediction models complicates the effective assessment of predictive performance differences among these models. Consequently, this study employs varying thresholds to compute the recall rate for the models being considered. The methodology involves designating the first k long noncoding RNAs (lncRNAs) associated with each disease as positive samples, while the remaining samples are classified as negative samples, facilitating the calculation of the overall recall rate. The findings are shown in Figure 4. The results indicate that the MVIGCN model is consistently superior to the other models across different values of k. When k is 30, 90, 150, and 210, the MVIGCN model achieves predicted recall rates of 71.15%, 89.54%, 96.55%, and 98.96%, respectively. In comparison, the MHRWR model yields top-k recall rates of 32.22%, 52.76%, 80.42%, and 99.00%; the GANLDA model yields recall rates of 47.87%, 76.94%, 94.55%, and 98.89%; the SIMACLDA model presents rates of 41.49%, 74.34%, 94.59%, and 99.90%; and the GAMCLDA model achieves top-k recall rates of 44.61%, 79.09%, 91.99%, and 97.74%. These experimental results indicate that the MVIGCN model is better able to identify associations between potential lncRNAs and diseases than the other models evaluated.

### 3.2 Case study

To investigate the capacity of the MVIGCN model to recognize the associations between lncRNAs and diseases in detail, this study designed relevant case study experiments. Within the dataset, all known associations are classified as positive samples, whereas all unknown relationships are treated as candidate associations. The analysis of these candidate associations ascertain whether the model can successfully identify previously unknown associations. This study focuses on several prevalent diseases, including breast cancer, liver cancer, and kidney cancer, as subjects for case studies. The methodological approach is as follows: initially, the model predicts all unknown associations. For example, under conditions of breast cancer, the correlation scores between candidate lncRNAs and breast cancer are ranked from highest to lowest. The top 20 lncRNAs were then validated against relevant databases and studies, which demonstrated that the candidate lncRNA‒disease associations predicted by the model provide valuable insights for guiding biological wet laboratory experiments.

The lncRNA–disease relationship data utilized in this research are sourced from LncRNADisease and Lnc2Cancer, as detailed in the supplement document, encompassing 2,697 relationships among 240 lncRNAs and 412 diseases. The remaining 96,183 unknown relations are considered candidate samples. In the process of validating candidate associations, this study sought relevant experimental evidence from Lnc2Cancer 3.0 and MNDR v3.1. In instances where pertinent data are not available in these three databases, the study then uses the National Center for Biotechnology Information (NCBI) database to obtain the results. Three diseases—breast cancer, lung cancer, and cervical cancer—were selected as the focus of the case studies. The subsequent sections analyse and present the findings from the case studies pertaining to these three diseases.

Breast cancer is the most prevalent malignancy among women and poses a significant threat to their health and wellbeing. Even when sex is not considered, breast cancer remains the second most common type of cancer following lung cancer; however, owing to its better prognosis, it ranks fifth in terms of the key cause of cancer-related mortality. In less developed regions, breast cancer is a typical cancer, with incidence rates significantly exceeding those in developed areas. Recent research has highlighted the critical role of lncRNAs in the onset and development of various diseases. For example, Zhang et al. (2024) reported that LINC00963 is related to the metastasis and development of breast cancer cells. Silencing LINC00963 expression has been shown to inhibit breast cancer progression. Furthermore, Zhang et al. (2024) demonstrated that knockout of the ACK1 gene diminishes the capacity of LINC00963 to promote breast tumour growth. These findings indicate that LINC00963 inhibits ACK1 activity by downregulating miR-324-3p expression, facilitating the development and metastasis of breast cancer. Consequently, LINC00963 may serve as a promising therapeutic target for breast cancer therapy.

Table 2 shows the results of the association verification for the top 20 candidate lncRNAs identified by the MVICGN model in the context of breast cancer. The findings indicate that 17 out of the 20 candidate lncRNAs have been validated by existing databases. Notably, SNHG1 has been identified as a significant regulator that facilitates tumorigenesis, and its expression levels are markedly elevated in breast cancer cells. Research conducted by Xiong et al. demonstrated that SNHG1 suppresses the expression of associated normal mRNAs and proteins in breast cancer cells through its interaction with miR-573 and simultaneously promotes the expression of relevant cyclins (Xiong et al., 2020). These observations imply that SNHG1 can be considered a promising target for intervention and prognostic assessment in breast cancer.

**TABLE 2:** Validation results of MVIGCN in predicting the top 20 candidate lncRNAs in breast cancer.

Rank	lncRNA	The verification results
1	MIR17HG	PMID: 25680407
2	BANCR	Lnc2Cancer 3.0, MNDR v3.1
3	TUG1	Lnc2Cancer 3.0, MNDR v3.1
4	DANCR	Lnc2Cancer 3.0, MNDR v3.1
5	HNF1A-AS1	PMID: 32319789
6	SNHG1	Lnc2Cancer 3.0, MNDR v3.1
7	HULU	Lnc2Cancer 3.0, MNDR v3.1
8	PCAT1	Lnc2Cancer 3.0, MNDR v3.1
9	NPTN-IT1	MNDR v3.1
10	LINC00261	PMID: 32440206
11	PRNCR1	Lnc2Cancer 3.0, MNDR v3.1
12	MYCNOS	Unknown
13	GHET1	Lnc2Cancer 3.0, MNDR v3.1
14	WT1-AS	Lnc2Cancer 3.0, MNDR v3.1
15	CRNDE	Lnc2Cancer 3.0、 LncRNADisease v2、 MNDR v3.1
16	MIR7-3HG	Unknown
17	HAND2-AS1	Lnc2Cancer 3.0, MNDR v3.1
18	LINC00473	Lnc2Cancer 3.0, MNDR v3.1
19	C1QTNF9B-AS1	Unknown
20	SCHLAP1	PMID: 34268927

Lung cancer represents the most prevalent and lethal form of cancer worldwide. Among its various types, NSCLC constitutes the majority, accounting for 85% of newly diagnosed lung cancer patients. This subtype is highly resistant to chemotherapy, resulting in poor prognoses for affected patients after treatment. LncRNAs are key regulators of the pathogenesis and progression of lung cancer. Research conducted by Tong et al. has demonstrated that the lncRNA CASC11 and the gene CDK1 are markedly overexpressed in lung cancer tissues, whereas microRNA-302 levels are decreased in these tissues. Experimental evidence suggests that CASC11 facilitates the progression of lung cancer by interacting with microRNA-302, increasing the expression of CDK1 (Tong et al., 2019).

Table 3 presents the top 20 candidate lncRNAs associated with lung cancer, as predicted by the MVIGCN model. The findings indicate that 17 out of the 20 candidate lncRNAs were corroborated by existing databases. For example, the candidate lncRNA HULC has been shown in various studies to be significantly elevated in the serum of lung cancer patients, with its concentration increasing in correlation with cancer progression. Research has indicated that the lncRNA HULC facilitates the expression of SPHK1, which accelerates the proliferation of NSCLC cells but inhibits their apoptosis. Furthermore, Jin et al. identified ZEB1-AS1 as a candidate lncRNA, ZEB1-AS1, with clinical therapeutic and prognostic significance for NSCLC patients (Jin et al., 2019). Investigations revealed that ZEB1-AS1 contributes to carcinogenesis by downregulating the expression of the ID1 gene in NSCLC cells. These results indicate that ZEB1-AS1 is a promising therapeutic target for NSCLC. Notably, the MVIGCN model also identified three previously unrecognized potential lncRNAs—NALT1, MIR100HG, and PISRT1—offering avenues for validation through biological wet experiments.

**TABLE 3:** Validation results of MVIGCN in predicting the top 20 candidate lncRNA in lung cancer.

Rank	lncRNA	The verification results
1	HOTTIP	Lnc2Cancer 3.0、 LncRNADisease v2 MNDR v3.1
2	HULC	Lnc2Cancer 3.0
3	TINCR	Lnc2Cancer 3.0、 LncRNADisease v2 MNDR v3.1
4	GHET1	Lnc2Cancer 3.0
5	NALT1	Unknown
6	TP53COR1	LncRNADisease v2、 MNDR v3.1
7	TUSC7	Lnc2Cancer 3.0、 MNDR v3.1
8	SPRY4-IT1	Lnc2Cancer 3.0、 LncRNADisease v2、 MNDR v3.1
9	MIR155HG	PMID: 32432745
10	MIR100HG	Unknown
11	ZFAS1	Lnc2Cancer 3.0
12	SOX2-OT	LncRNADisease v2、 MNDR v3.1
13	CBR3-AS1	PMID: 32945466
14	KCNQ1OT1	Lnc2Cancer 3.0、 LncRNADisease v2 MNDR v3.1
15	HOTAIRM1	Lnc2Cancer 3.0、 MNDR v3.1
16	PANDAR	Lnc2Cancer 3.0、 MNDR v3.1
17	DANCR	Lnc2Cancer 3.0、 LncRNADisease v2 MNDR v3.1
18	PISRT1	Unknown
19	HOXA11-AS	Lnc2Cancer 3.0
20	ZEB1-AS1	Lnc2Cancer 3.0

Cervical cancer ranks among the most prevalent gynaecological malignancies, which justifies its selection as the focus of the third case study. The findings are presented in Table 4. Among the top 20 candidate lncRNAs related to cervical cancer, 14 predictive relationships were identified. For example, research conducted by Zhu et al. revealed that the mRNA expression of the candidate lncRNA CDKN2B-AS1 was significantly upregulated in cervical cancer cells, whereas the expression of miR-181a-5p was notably downregulated in cervical cancer cells. Subsequent experiments demonstrated that CDKN2B-AS1 facilitates cervical cancer cell proliferation but inhibits their senescence. This body of research indicates that CDKN2B-AS1 has a key role in disease onset and development through its interaction with miR-181a-5p. Additionally, investigations by Shen et al. revealed that the expression level of the candidate lncRNA MIR155HG was markedly elevated in cervical cancer tissues compared with normal cervical tissues. Notably, knockout of the MIR155HG gene resulted in the inhibition of cervical cancer cell proliferation, suggesting that MIR155HG is involved in the pathogenesis of cervical cancer and may serve as a valuable therapeutic target (Shen et al., 2020).

**TABLE 4:** Validation results of MVIGCN in predicting the top 20 candidate lncRNA in cervical cancer.

Rank	lncRNA	The validation results
1	UCA1	Lnc2Cancer 3.0、 LncRNADisease v2 MNDR v3.1
2	NEAT1	Lnc2Cancer 3.0、 LncRNADisease v2 MNDR v3.1
3	LINC00961	Unknown
4	LSINCT5	Unknown
5	CDKN2B-AS1	Lnc2Cancer 3.0、 LncRNADisease v2 MNDR v3.1
6	CCAT1	Lnc2Cancer 3.0、 LncRNADisease v2 MNDR v3.1
7	TUG1	Lnc2Cancer 3.0、 LncRNADisease v2 MNDR v3.1
8	LUCAT1	Lnc2Cancer 3.0、 MNDR v3.1
9	MIR17HG	PMID: 32920922
10	HNF1A-AS1	Lnc2Cancer 3.0、 MNDR v3.1
11	HOXA11-AS	Lnc2Cancer 3.0、 LncRNADisease v2 MNDR v3.1
12	HOTTIP	Unknown
13	HYMAI	PMID: 34120615
14	NPSR1-AS1	Unknown
15	LINCMD1	Unknown
16	LINC00271	Unknown
17	XIST	Lnc2Cancer 3.0、MNDR v3.1
18	AFAP1-AS1	Lnc2Cancer 3.0、MNDR v3.1
19	SNHG12	Lnc2Cancer 3.0、 LncRNADisease v2 MNDR v3.1
20	MIR155HG	PMID: 33262605

## 4 Conclusion

This research presents the MVIGCN model to predict the relationships between lncRNAs and diseases. Initially, we provide an overview of the dataset utilized in this model. Subsequently, we detail the methodology for constructing the lncRNA-related network, with various approaches for calculating similarities between lncRNAs and diseases. We elaborate on the construction of the input feature vector of the model, the computation methods employed in the graph CNN layer, the GAT, the model decoder, and the loss function used during model training. We conduct a comparative analysis of the MVIGCN model against the GAMCLDA, GANLDA, MHRWR, and SIMCLDA models through experimental validation. The predictive property of the model is assessed via 50%fold cross-validation, and we compare the AUC, AUPR, F1 score, MCC score, and top-k metric across the same dataset. The results indicate that the MVIGCN model is superior to the other models. Moreover, we establish ablation experiments, and in a case study, we demonstrated that the MVIGCN model is capable of predicting potential lncRNAs associated with prevalent cancers: breast cancer.

Research has shown that lncRNAs often regulate physiological processes through complex networks composed of various biomolecules such as miRNAs and proteins. The MVIGCN model accurately predicts deep associations between lncRNAs and diseases based on the regulatory networks of these biomolecules, thereby addressing the problem of isolated nodes.

Specifically, the model first constructs an lncRNA-miRNA-disease heterogeneous network and an lncRNA-disease association network. Then, it uses Graph Convolutional Neural Networks (GCNs) and Graph Attention Networks (GATs) to extract structural features from the regulatory networks, respectively, and fuses the features from both networks to ultimately predict lncRNA-disease associations. Compared to other algorithms, the MVIGCN model has two significant advantages. First, considering that lncRNA-miRNA-disease associations are important pathways for lncRNA function, the model incorporates regulatory information about miRNAs, and ablation experiments demonstrate the effectiveness of this approach. Second, by simultaneously using GCNs and GATs to extract network features and fusing multiple regulatory relationships, the MVIGCN model outperforms other models in five-fold cross-validation experiments.

## Data Availability

The datasets presented in this study can be found in online repositories. The names of the repository/repositories and accession number(s) can be found in the article/Supplementary Material.
