# Supplementary material for: Association prediction of lncRNAs and diseases using multiview graph convolution neural network
Source: Front Genet. 2025 Apr 15;16:1568270. doi: 10.3389/fgene.2025.1568270 (PMC12037633; doi:10.3389/fgene.2025.1568270)
Supplement: Supplementary file 1 [file Supplementaryfile1.docx]

Supplementary Material

**Wei Zhang**1**, Yifu Zeng**1**, Xiaowen Xiang**1**, Bihai Zhao**1**, Sai Hu^2^, limiao Li**1**, Xiaoyu Zhu**1**, and Lei Wang**1,*

1 College of Computer Science and Engineering, Changsha University, Changsha, Hunan 410022, China

2 Department of Information and Computing Science, College of Mathematics, Changsha University, 410003 Changsha, China

*[corresponding. wanglei@xtu.edu.cn](mailto:corresponding.author@email.example)

# Data introduction

The MVIGCN model is predicated on the regulatory networks of biological macromolecules, specifically lncRNAs, and their relationships with various diseases. This model requires several types of data, including lncRNA‒disease association data, as well as disease and lncRNA similarity data. The statistical details regarding the data sources, sample sizes, and number of associations are presented in Table 1. The lncRNA–disease relationship data are sourced from the LncRNADisease and LncCancer databases, encompassing a total of 2,697 associations involving 412 diseases and 240 lncRNAs. This relationship is represented in a 240 × 412 matrix (denoted LD), where as indicated in Equation (1), a value of 1 in the matrix signifies a known relationship between disease $i$ and lncRNA $j$, whereas a value of 0 indicates an unknown association. The lncRNA‒miRNA relationship data were obtained from the StarBase v2.0 database, which includes 1,002 associations between 240 lncRNAs and 495 miRNAs. The association matrix for lncRNA‒miRNA (denoted LM) is defined similarly, as shown in Equation (2). Furthermore, the miRNA‒disease relationship data are derived from HMDD v2.0, which contains 13,562 relationships between diseases and miRNAs, with the corresponding association matrix (MD) defined in Equation (3). Additionally, the MVIGCN model incorporates data on functional similarities among diseases and lncRNAs.

**Table 1.** Statistics of relevant data sources

| **Types** | **Number of samples** | **Number of associations** | **data sources** |
| --- | --- | --- | --- |
| LncRNA-Disease | $240\times412$ | 2697 | LncRNADisease、Lnc2Cancer |
| LncRNA-miRNA | $240\times495$ | 1002 | StarBase v2.0 |
| miRNA-Disease | $495\times412$ | 13562 | HMDD v2.0 |
| Disease-Disease | $412\times412$ | - | Disease Ontology 2015 |

$$\begin{aligned} {LD}_{ij}=\left\{ \begin{aligned} 1, &{lncRNA}_{i}\mathrm{and}{Disease}_{j} Known associated \\ 0, &{lncRNA}_{i}\mathrm{and}{Disease}_{j} Unknown associated \end{aligned} \right.\#(1) \end{aligned}$$

$$\begin{aligned} {LM}_{ij}=\left\{ \begin{aligned} 1, &{lncRNA}_{i}\mathrm{and}{miRNA}_{j} Known associated \\ 0, &{lncRNA}_{i}\mathrm{and}{miRNA}_{j} Unknown associated \end{aligned} \right.\#(2) \end{aligned}$$

$$\begin{aligned} {MD}_{ij}=\left\{ \begin{aligned} 1, &{miRNA}_{i}\mathrm{and}{Disease}_{j} Known associated \\ 0, &{miRNA}_{i}\mathrm{and}{Disease}_{j} Unknown associated \end{aligned} \right.\#(3) \end{aligned}$$

# Methodology for Similarity Calculation and Network Construction

1. Development of a Three-Layer Correlation Network Involving lncRNAs, miRNAs, and Disease

The MVIGCN prediction model is predicated on the fundamental hypothesis that similar lncRNAs are likely to be associated with similar diseases. This model extracts topological structural characteristics from various biological macromolecule association networks, facilitating the learning of both direct and indirect relationships between lncRNAs and diseases for the purpose of predicting their interrelations. Consequently, the initial phase of this model involves the construction of a robust heterogeneous network that links lncRNAs and diseases, which provides reliable data for the association prediction framework.

lncRNAs are integral to biological processes. In recent years, miRNAs, which are also noncoding RNAs capable of regulating human physiological and pathological processes, have garnered increased attention from researchers regarding their mechanisms of action and regulatory relationships, particularly in comparison to those of lncRNAs. Like lncRNAs, miRNAs are implicated in the onset and development of various diseases. Ongoing research efforts have led to the identification of a diverse array of miRNA mechanisms, alongside the establishment of multiple data resources, including miRNA–disease relation databases and miRNA–lncRNA relation databases. Furthermore, lncRNAs and miRNAs can mutually regulate, influencing the development of cancer and other diseases. Given the intricate and multifaceted regulatory mechanisms associated with lncRNAs, it is imperative to utilize a variety of biological data to thoroughly investigate the relationships between lncRNAs and diseases, improving the predictive precision of the model.

**Figure 1.** Illustrates a three-layer correlation network involving lncRNAs, miRNAs, and diseases.

By utilizing comprehensive miRNA-related data resources, MVIGCN initially investigated the associations among miRNAs. This study builds on the foundational hypothesis that "similar lncRNAs may be related to similar diseases" by proposing that "lncRNAs associated with the same miRNA may be linked to the same disease". As shown in Figure 1, a three-layer association network comprising lncRNAs and diseases, which is referred to as [net]_1, was constructed. The three distinct layers depicted in the figure correspond to lncRNAs, miRNAs, and diseases. The connection between nodes in the two layers of networks indicates that a known relationship exists between the two nodes. Otherwise, the association between the two nodes is unknown. As shown in Equation (4), the adjacency matrix $A_{1}$ of ${net}_{1}$ is defined as follows, where LM and MD refer to the lncRNA‒miRNA incidence matrix and miRNA-disease incidence matrix, respectively, ${LM}^{T}$ and ${MD}^{T}$ represent the transpose matrix of the LM matrix and MD matrix, respectively, and O represents the all-0 matrix.

$$\begin{aligned} A_{1}=\left[ \begin{matrix} I & LM & O \\ {ML}^{T} & I & MD \\ O & {MD}^{T} & I \end{matrix} \right]\#\left( 4 \right) \end{aligned}$$

2. Construction of a Two-Layer Heterogeneous Network for lncRNA‒Disease Associations

This study is predicated on the fundamental premise that "functionally analogous lncRNAs may be linked to diseases exhibiting similar phenotypic characteristics." Hence, a two-layer heterogeneous network encompassing lncRNA‒disease associations has been developed, integrating a functional similarity network for lncRNAs, a phenotypic similarity network for diseases, and a lncRNA‒disease association network, as shown in Figure 2. With this network, the MVIGCN model employs a graph deep learning algorithm to extract pertinent information regarding the functional similarities among lncRNAs and the phenotypic similarities among diseases. This approach facilitates the exploration of the relationships between diseases and lncRNAs from a similarity perspective. The implementation of appropriate computational methodologies for assessing lncRNA and disease similarities is crucial for addressing the challenges posed by isolated diseases and lncRNAs, as well as for enhancing the predictive performance of the model. The subsequent sections detail the methodologies employed for calculating disease and lncRNA similarities, as well as the procedural steps involved in constructing the lncRNA‒disease two-layer heterogeneous network.

**Figure 2.**lncRNA- disease two-layer network

1. The computational framework for assessing semantic similarity in disease classification.

Disease A is expressed as the graph ${DAG}_{A}=\left( A，T_{A}，E_{A} \right)$, where $A$, $T_{A}$, and $E_{A}$ represent the ancestor nodes and associated edges of diseases A and A, respectively. First, the semantic contribution value $D_{A}\left( t \right)$ of disease t and disease A in ${DAG}_{A}$, is defined and calculated as follows ^1^:

$$\begin{aligned} \left\{ \begin{aligned} &D_{A}\left( t \right)=1 if t=A \\ &D_{A}\left( t \right)=max\left\{ \Delta*D_{A}\left( t^{'} \right)|t^{'}\in children of t \right\} if t\neq A \end{aligned} \right.\#\left( 5 \right) \end{aligned}$$

where ∆ denotes the semantic contribution parameter associated with disease t and the corresponding child disease $t^{'}$. Disease A is the most specific disease classification, so the semantic contribution value of A to itself is 1. A node farther from disease A is a more generalized disease classification, and its semantic contribution value should also be lower. Thus, the value interval of ∆ should be (0, 1). On the basis of Equation (5), the semantic value of disease A can be calculated via the following formula ^2^:

$$\begin{aligned} DV\left( A \right)=\sum_{t\in T_{A}} D_{A}\left( t \right)\#\left( 6 \right) \end{aligned}$$

As mentioned above, assuming that two diseases with more common ancestor nodes have higher semantic similarity, the semantic relevance of the disease can be determined by the relative position of the disease in the disease DAG. The semantic similarity of diseases A and B is defined as follows ^3^:

$$\begin{aligned} DSS\left( A，B \right)=\frac{\sum_{t\in T_{A}\cap T_{B}} \left( D_{A}\left( t \right)+D_{B}\left( t \right) \right)}{DV\left( A \right)+DV\left( B \right)}\#\left( 7 \right) \end{aligned}$$

where $DSS\left( A，B \right)$ represents the semantic similarity, $DV\left( A \right)$ and $DV\left( B \right)$ represent the semantic values, and $D_{A}\left( t \right)$ and $D_{B}\left( t \right)$ represent the semantic contribution values of disease t to diseases A and B, respectively. On the basis of the above method, this paper uses the DincRNA online toolkit ^4^ to determine the semantic similarity in 412 diseases and obtains the DSS matrix of 412 diseases. The entity $DSS\left( i,j \right)$ represents the semantic similarity value in disease *i* and disease *j*.

1. the Calculation model of lncRNA functional similarity.

According to the basic hypothesis that lncRNAs related to similar disease sets have similar functions, the functional similarity of lncRNAs was determined via the disease sets associated with them. This paper refers to the calculation model proposed by Chen et al. ^5^ to calculate the functional similarity. Using the calculation of the functional similarity of lncRNAs $u$ and $v$ as an example, we define $DG\left( u \right)$ as the set of lncRNA $u$-related diseases, define $DG\left( u \right)=\left\{ d\left( u1 \right),d\left( u1 \right),\ldots,d\left( um \right) \right\}$ and define DG (v) as the set of lncRNA v-related diseases, define $DG\left( v \right)=\left\{ d\left( v1 \right),d\left( v1 \right),\ldots,d\left( vn \right) \right\}$, and use the similarity between $D\left( u \right)$ and $D\left( v \right)$ as the functional similarity of lncRNAs $u$ and $v$. To determine the similarity between $D\left( u \right)$ and $D\left( v \right)$, first, the similarity between a related disease of one lncRNA and the related disease set of another lncRNA is calculated. For example, the similarity calculation formula between the disease $d\left( u1 \right)$ related to lncRNA $u$ and the disease set $DG\left( v \right)$ related to lncRNA $v$ is as follows ^6^:

$$\begin{aligned} S\left( d\left( u1 \right)，DG\left( v \right) \right)=\max_{d\in DG\left( v \right)} \left( SS\left( d\left( u1 \right),d \right) \right)\#\left( 8 \right) \end{aligned}$$

The similarity between $DG\left( u \right)$ and $DG\left( v \right)$ of the disease set is subsequently calculated as follows ^7^:

$$\begin{aligned} LFS\left( u,v \right)=\frac{\sum_{i=1}^{m} S\left( d\left( ui \right),DG\left( v \right) \right)+\sum_{j=1}^{n} S\left( DG\left( u \right),d\left( vi \right) \right)}{m+n}\#\left( 9 \right) \end{aligned}$$

where m and n refer to the number of disease sets related to lncRNAs $u$ and $v$, respectively, and $LFS\left( u,v \right)$ represents the functional similarity. According to the above calculation method, the matrix $LFS$ for 240 lncRNAs is obtained, and the entity $LFS\left( i,j \right)$ refers to the functional similarity value of two lncRNAs.

1. A computational model of kernel similarity for diseases.

This research applied the model proposed by Chen et al. ^5^ to determine the GIP similarity. Using the calculation of the similarity in disease $d\left( i \right)$ and $d\left( j \right)$ GIP as an example, we define the interaction spectrum $IP\left( d\left( i \right) \right)$ of the *i*-th behaviour disease 𝑑𝑖 of the lncRNA-disease relation matrix 𝐴. $IP\left( d\left( i \right) \right)$ is a binary vector representing the correlation in $d\left( i \right)$ and each lncRNA. Similarly, we define the interaction spectrum $IP\left( d\left( j \right) \right)$ of disease $d\left( i \right)$. On the basis of the disease interaction spectrum described above, the similarity between $d\left( i \right)$ and $d\left( j \right)$ is expressed as follows:

$$\begin{aligned} DGIP\left( d\left( i \right)，d\left( j \right) \right)=exp\left( -\gamma_{d}\left\| IP\left( d\left( i \right) \right)-IP\left( d\left( j \right) \right) \right\|^{2} \right)\#\left( 10 \right) \end{aligned}$$

$$\begin{aligned} \gamma_{d}=\frac{\gamma_{d}^{'}\left( \sum_{i=1}^{nd} \left\| IP\left( d\left( i \right) \right) \right\|^{2} \right)}{nd}\#\left( 11 \right) \end{aligned}$$

where $nd$ represents the total number of diseases and $\gamma_{d}$ is a hyperparameter that controls the width of the nucleus. Its value is related to the average number of disease-associated lncRNAs and is calculated by another normalized nuclear bandwidth hyperparameter $\gamma_{d}^{'}$. According to experience, the value of $\gamma_{d}^{'}$ is often 1, and the calculation method of $\gamma_{d}$ is shown in Equation (11). Thus, the similarity matrix DGIP for 412 diseases is obtained, and the entity $DGIP(i,j)$ represents the similarity value in disease $d\left( i \right)$ and disease $d\left( j \right)$.

1. Kernel similarity calculation model of lncRNAs.

As mentioned above, the kernel similarity of lncRNAs is also computed in this paper on the basis of the computational model constructed by Chen et al. ^5^. The calculation formula is as follows:

$$\begin{aligned} LGIP\left( l\left( i \right)，l\left( j \right) \right)=exp\left( -\gamma_{d}\left\| IP\left( l\left( i \right) \right)-IP\left( l\left( j \right) \right) \right\|^{2} \right)\#\left( 12 \right) \end{aligned}$$

$$\begin{aligned} \gamma_{l}=\frac{\gamma_{l}^{'}\left( \sum_{i=1}^{nd} \left\| IP\left( l\left( i \right) \right) \right\|^{2} \right)}{nl}\#\left( 13 \right) \end{aligned}$$

where $IP\left( l\left( i \right),l\left( j \right) \right)$ is the similarity value of the kernel between $l\left( i \right)$ and $l\left( j \right)$, $IP\left( l\left( i \right) \right)$ and $IP\left( l\left( j \right) \right)$ are the interaction kernels of $l\left( i \right)$ and $l\left( j \right)$, respectively, and $\gamma_{l}$ is the bandwidth regulator, which is also related to the mean number of diseases related to the lncRNA. $nl$ refers to the number of lncRNAs, and $\gamma_{l}^{'}$ is the normalized nuclear bandwidth regulator, which is usually set to 1 according to experience. The kernel similarity $LGIP$ in 240 lncRNAs is obtained, and the entity $LGIP(i,j)$ represents the kernel similarity value.

1. Calculation model of disease cosine similarity.

In multidimensional space, the relationship between each disease and other biological macromolecules can be expressed as a one-dimensional vector, and the similarity between two vectors can be used to represent the similarity in diseases. In this work, the correlation between a disease and a lncRNA is used to represent the vector corresponding to the disease, and the cosine similarity of the vectors of the two diseases is regarded as the similarity of different diseases. The calculation method is as follows:

$$\begin{aligned} DCOS\left( d\left( i \right),d\left( j \right) \right)=\frac{LD\left( :,i \right)\cdot LD\left( :,j \right)}{\left\| LD\left( :,i \right) \right\|\times\left\| LD\left( :,j \right) \right\|}\#\left( 14 \right) \end{aligned}$$

where $DCOS\left( d\left( i \right),d\left( j \right) \right)$ represents the cosine similarity in $d\left( i \right)$ and $d\left( j \right)$; $LD$ is the offset matrix; $LD\left( :,i \right)$ represents the i-th column of matrix $LD$, that is, the corresponding one-dimensional vector of $d\left( i \right)$; and $LD\left( :,j \right)$ is the corresponding one-dimensional vector of disease $d\left( j \right)$. $DCOS$ is the cosine similarity matrix among 412 diseases, in which the value range of the elements is [0, 1].

1. Calculation Model of the Cosine Similarity of lncRNAs.

As previously mentioned, to integrate multiple types of similarity information widely, this paper also introduces cosine similarity. The calculation method is as follows:

$$\begin{aligned} LCOS\left( l\left( i \right),l\left( j \right) \right)=\frac{LD\left( i,: \right)\cdot LD\left( j,: \right)}{\left\| LD\left( i,: \right) \right\|\times\left\| LD\left( j,: \right) \right\|}\#\left( 15 \right) \end{aligned}$$

where $LCOS\left( l\left( i \right),l\left( j \right) \right)$ is the cosine similarity between $l\left( i \right)$ and $l\left( j \right)$; $LD\left( i,: \right)$ represents the i-th line of matrix $LD$, that is, the corresponding one-dimensional vector of $l\left( i \right)$; and $LD\left( :,j \right)$ is the corresponding vector of disease $l\left( j \right)$. $LCOS$ is a cosine similarity matrix between 240 lncRNAs.

As mentioned above, the calculation of kernel similarity and cosine similarity between diseases and lncRNAs is based on the lncRNA‒disease relationship. Therefore, this paper uses different weights to fuse the two similarities between diseases and lncRNAs. The vector-based similarity calculation formula for disease-related lncRNAs is as follows:

$$\begin{aligned} LGCS=\alpha_{1}\cdot LGIP+\alpha_{2}\cdot LCOS \left( 0.1<\alpha_{1},\alpha_{2}<0.9 \right)\#\left( 16 \right) \end{aligned}$$

$$\begin{aligned} DGCS=\beta_{1}\cdot DGIP+\beta_{2}\cdot DCOS \left( 0.1<\beta_{1},\beta_{2}<0.9 \right)\#\left( 17 \right) \end{aligned}$$

where $LGCS$ and $DGCS$ represent the similarity based on the vector distance measurement; $\alpha_{1}$ and $\alpha_{2}$ represent the weight values of the kernel similarity value and cosine similarity value of the lncRNA, respectively; and $\beta_{1}$ and $\beta_{2}$ represent the weight values of two kinds of similarity of diseases, respectively. With reference to the experimental experience of Fan et al. ^8^, when the $\alpha_{1}$, $\alpha_{2}$, $\beta_{1}$ and $\beta_{2}$ values are 0.1, 0.9, 0.2 and 0.8, respectively, in the testing dataset used in the present research, the model has the best performance.

The functional similarity between lncRNAs and diseases and the similarity based on vector distance were fused. Inspired by Chen et al., the final comprehensive similarity calculation formula for lncRNAs is as follows:

$$\begin{aligned} LS\left( l\left( i \right),l\left( j \right) \right)=\left\{ \begin{aligned} LFS\left( l\left( i \right),l\left( j \right) \right) & if LFS\left( l\left( i \right),l\left( j \right) \right)\neq0 \\ LGCS\left( l\left( i \right),l\left( j \right) \right) & else LFS\left( l\left( i \right),l\left( j \right) \right)=0 \end{aligned} \right.\#\left( 18 \right) \end{aligned}$$

where $LS$ is the comprehensive similarity matrix of lncRNAs and $LS\left( l\left( i \right),l\left( j \right) \right)$ represents the comprehensive similarity value between $l\left( i \right)$ and $l\left( j \right)$.

$$\begin{aligned} DS\left( d\left( i \right),d\left( j \right) \right)=\left\{ \begin{aligned} DFS\left( d\left( i \right),d\left( j \right) \right) & if DFS\left( d\left( i \right),d\left( j \right) \right)\neq0 \\ DGCS\left( d\left( i \right),d\left( j \right) \right) & else DFS\left( d\left( i \right),d\left( j \right) \right)=0 \end{aligned} \right.\#\left( 19 \right) \end{aligned}$$

where $DS$ is the comprehensive similarity matrix of the disease and $DS\left( d\left( i \right),d\left( j \right) \right)$ refers to the comprehensive similarity value between $d\left( i \right)$ and $d\left( j \right)$. Therefore, the bias matrix $A_{2}$ of the two-layer heterogeneous network ${net}_{2}$ of lncRNA diseases is as follows:

$$\begin{aligned} A_{2}=\left[ \begin{matrix} LS & LD \\ {LD}^{T} & DS \end{matrix} \right]\#\left( 20 \right) \end{aligned}$$

REFERENCES

1 Xie, Y., Zhang, Y., Gong, M., Tang, Z. & Han, C. MGAT: Multi-view Graph Attention Networks. *Neural networks : the official journal of the International Neural Network Society* **132**, 180-189, doi:10.1016/j.neunet.2020.08.021 (2020).

2 Zheng, K. *et al.* Line graph attention networks for predicting disease-associated Piwi-interacting RNAs. *Briefings in bioinformatics* **23**, doi:10.1093/bib/bbac393 (2022).

3 Wang, D., Wang, J., Lu, M., Song, F. & Cui, Q. Inferring the human microRNA functional similarity and functional network based on microRNA-associated diseases. *Bioinformatics (Oxford, England)* **26**, 1644-1650, doi:10.1093/bioinformatics/btq241 (2010).

4 Cheng, L., Hu, Y., Sun, J., Zhou, M. & Jiang, Q. DincRNA: a comprehensive web-based bioinformatics toolkit for exploring disease associations and ncRNA function. *Bioinformatics* **34**, 1953-1956, doi:10.1093/bioinformatics/bty002 (2018).

5 Chen, X. *et al.* Constructing lncRNA functional similarity network based on lncRNA-disease associations and disease semantic similarity. *Scientific reports* **5**, 11338, doi:10.1038/srep11338 (2015).

6 Chen, X. *et al.* Computational models for lncRNA function prediction and functional similarity calculation. *Briefings in functional genomics* **18**, 58-82, doi:10.1093/bfgp/ely031 (2019).

7 Li, Y. *et al.* iLncDA-RSN: identification of lncRNA-disease associations based on reliable similarity networks. *Front Genet* **14**, 1249171, doi:10.3389/fgene.2023.1249171 (2023).

8 Fan, Y., Chen, M. & Pan, X. GCRFLDA: scoring lncRNA-disease associations using graph convolution matrix completion with conditional random field. *Briefings in bioinformatics* **23**, doi:10.1093/bib/bbab361 (2022).
